# Bolivian River Dolphin trends: A long-term analysis in the Mamore basin

**DOI:** 10.1371/journal.pone.0308806

**Published:** 2024-10-04

**Authors:** Luis A. Guizada Duran, Enzo Aliaga-Rossel, Mariana Paschoalini Frias, Alexandre N. Zerbini

**Affiliations:** 1 Programa de Pós-Graduação em Biodiversidade e Conservação da Natureza, Universidade Federal Juiz de Fora, Juiz de Fora, Minas Gerais, Brazil; 2 Institute of Ecology, Universidad Mayor de San Andrés, La Paz, Bolivia; 3 World Wide Fund for Nature–Brazil, Brasília, BR; 4 Cooperative Institute for Climate, Ocean and Ecosystem Studies, University of Washington & National Marine Mammal Laboratory, Alaska Fisheries Science Center, NOAA Fisheries, Seattle, WA, United States America; 5 Marine Ecology and Telemetry Research, Seabeck, WA, United States America; 6 Institute Aquile, Juiz de Fora, Minas Gerais, Brazil; Central University of South Bihar, INDIA

## Abstract

South American river dolphins face significant threats from intense human activities, resulting in habitat loss, fragmentation of their natural connectivity, overfishing, pollution, and incidental and intentional catches for use as bait for fisheries. From 1998 to 2022, 12 surveys were conducted in a river system in the Mamore River (Ibare-Tijamuchi-Mamore) basin, one of the primary distribution areas of the Bolivian river dolphin (BRD ‐ *Inia geoffrensis boliviensis*). Generalized linear models (GLMs) were used to assess population trends. The most supported model does not definitively indicate a decline in population. The estimated mean annual rate of population change for BRDs over the 24-year monitoring period was -0.0115 per year. The average count of BRDs in the Ibare River is lower (mean = 20, n = 4) compared to the mean of Tijamuchi (mean = 260, n = 4), and the same pattern is observed with the Mamore River (mean = 76, n = 4). There is tentative visual evidence of negative trend for the count of BRD based on the GLM curves, but the statistics are still inconclusive to the sub-basin of the Mamore River. This study highlights the importance of continue with monitoring efforts on river dolphin populations. Similar population dynamics are observed in other river dolphin species in the Amazon region, requiring immediate actions to reduce mortality and reverse the concerning decreasing trend exhibited by these populations.

## Introduction

Defaunation during the Anthropocene has been considered a critical conservation issue globally [1]. The estimated global vertebrate extinction rate is 100 times greater than the average of the last ten million years and continues to accelerate [2, 3], the highest proportion of extinctions has occurred in freshwater ecosystems [4]. Similarly, trends in vertebrate population have been rapidly declining since 1970, with an 84% decrease in the diversity of freshwater species [3].

In most tropical areas people use natural resources having direct access to rivers and other freshwater systems [5]. These activities include fishing for food and commerce, irrigation, transportation, construction, industrial activities, recreation, and cultural relationship. Also involving habitat modifications such as hydroelectric dams to supply electricity, reducing flood risk, large-scale agro-industrial irrigation and improving transport. These events, however, greatly contribute to habitat degradation and destruction, limiting species range and generating unfavorable conditions to river dolphin-human interactions [6, 7].

River dolphins are a particularly vulnerable group of freshwater small cetaceans found in tropical rivers in Asia and South America [8]. As an example, the result of heavily fishery interactions and strong habitat degradation, the Baiji (*Lipotes vexilifer*) is functionally extinct [9], while the Gangetic and Indian river dolphins (*Platanista gangetica gangetica* and *P*.*g*. *minor*) face relatively fast populations decline, affected by changes habitat structure caused by anthropogenic activities [8, 10, 11].

Recent evidence suggests a similar scenario for the Amazon river dolphin (*Inia geoffrensis*) in Central Amazon, Brazil [6]. Therefore, all species of the genus *Inia* are listed as "Endangered" (EN) under the IUCN Red List, partly due to habitat degradation and modification, accidental mortality, and intentional catches, which have resulted in a concerning population decline. It is unlikely that these pressures will diminish unless there is a significant change in the socio-political, economic, and human demographic landscape [6].

The Bolivian River dolphin (BRD) *(Inia geoffrensis boliviensis*, d’Orbigny 1834) belongs to the Amazonian River dolphin group (Genus: Inia) and was naturally separated from *Inia geoffrensis* by rapids and cascades, a geographic barrier between Porto Velho and Guajará-Mirim in Brazil [6, 12]. Geographic isolation has prevented any genetic flow that could enhance the populations on the Bolivian side upriver [13]. The construction of Jirau and San Antonio dams in Brazil, have isolated the Amazonian river-dolphin population and most likely will have consequences in the area [14], however, these dams are located downriver, below the rapids and cascades and does not affect directly the upriver BRD populations in Bolivia.

On the other hand, despite significant scientific evidence [15, 16], the BRD has not yet been officially recognized as a distinct species by the Society for Marine Mammalogy Committee on Taxonomy [17]. Despite its isolation, is considered the least threatened species within the Amazonian dolphin group and holds a favorable conservation status [18].

Nevertheless, likewise other river dolphin species, the BRD is susceptible to the cumulative impacts of human-induced alterations in the freshwater environment. With a low reproductive rate and at least two-year birth interval, the BRD has limited ability to recover from any population loss [19, 20]. Despite Bolivia declared BRD as national natural heritage, and released a conservation action plan, there have been few endeavors to comprehend its population dynamics and to implement any direct conservation action. In this study, we investigated the population trend scenario of the BRD on its core distribution range in the middle Mamore River (Ibare-Mamore-Tijamuchi complex), Bolivia. Over the past 30 years, this region has been the major focus for BRD research, mainly due to the high level of human activity and constitutes the principal area of its distribution. Monitoring data collected over this time can now provide valuable insights to understanding the BRD’s population shifts, and possible factors contributing to its declining.

## Materials and methods

### Study area

**Fig 1:**
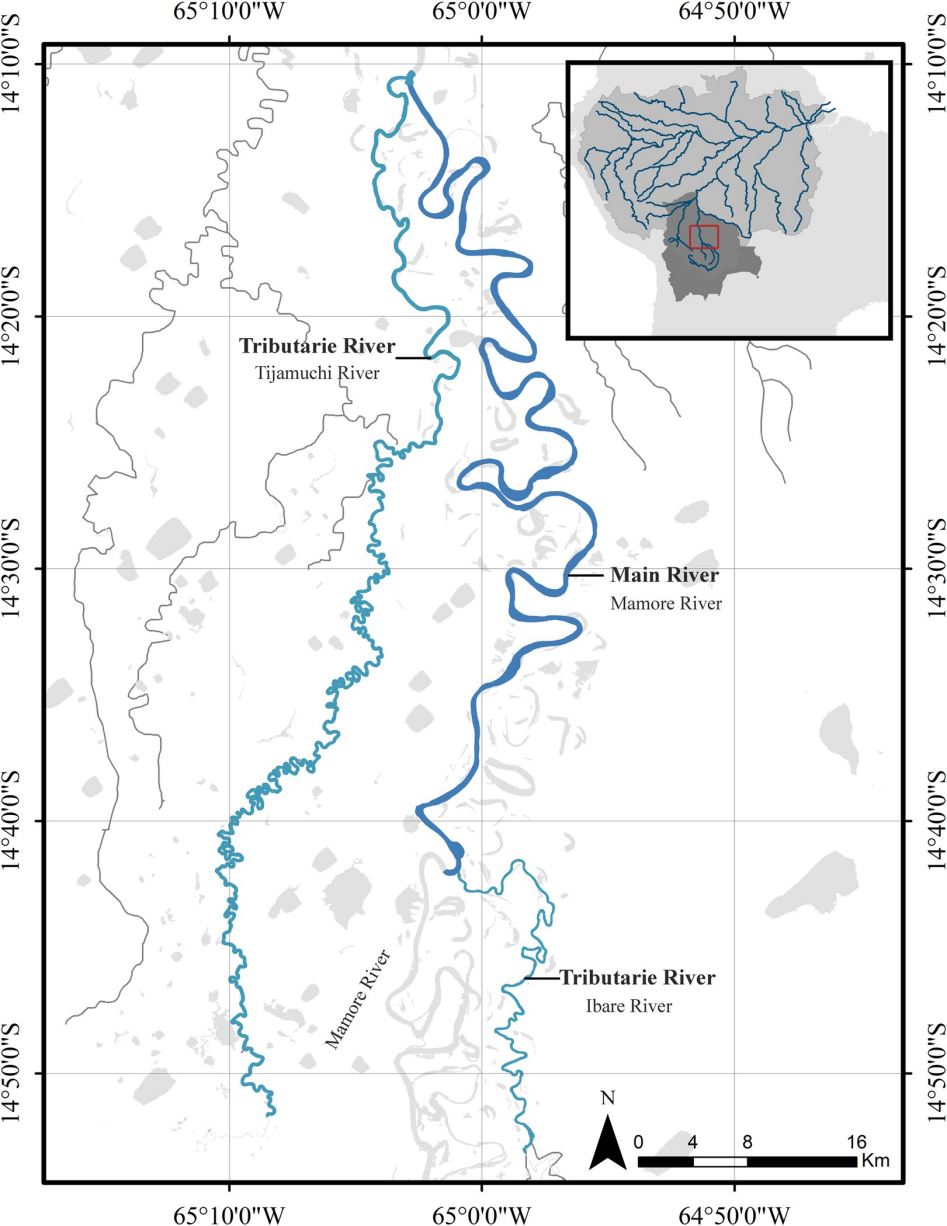
Area of study. Complex formed by three rivers: Ibare and Tijamuchi as tributaries and Mamore as the main river of the sub-basin in Bolivia.

The river dolphin abundance survey area is located in the Bolivian Amazon, in the Mamore River basin in the department of Beni, Bolivia. On the Ibare and Tijamuchi rivers, both clearwater tributaries of the Mamore River (Fig 1). These rivers exhibit clear water characteristics and form a meandering system with noticeable fluctuations in water levels corresponding to the low-water and high-water seasons.

During the high-water season, the average width of the tributary rivers (Ibare and Tijamuchi) is approximately 150 meters, whereas during the low-water season, it decreases to less than half that size, ranging from 50 to 70 meters. In contrast, the Mamore River, one of the main white-water rivers in Bolivia, maintains a width greater than 400 meters during the high-water season.

Besides hosting a significant population of BRD throughout the year, the central Mamore basin experiences substantial anthropogenic use and pressures resulting from various stressors. These stressors include intense boat traffic due to heavy vessels transportation, overfishing, strong deforestation, and frequent uncontrolled human induced forest fires, accidental BRD entanglement in nets (resulting in the mortality especially of calves and juveniles), poaching, and pollution [21, 22].

### Data collection

Trends in abundance of the BRD were estimated using dolphin counts on 12 independent visual boat-surveys (1998 to 2022) carried out during the transitional water period of rising and receding waters (i.e., between flooded and dry seasons). The transitional water period is when most of the riverine habitat types are available for river dolphins use, making its distribution less concentrated and increasing boat accessibility in shallow channels [23]. Data collections protocols and methods used were detailed described in Aliaga-Rossel [24] and Gomez-Salazar, Portocarrero-Aya [23]. It involved a combination of line and strip transect, with specific adaptations depending on factors such as the width of the river and the prevailing water level. The surveys were conducted at an average speed of 7–9 km/h on tributaries rivers and 12–15 km/h on Mamore River (due to the force of stream). While all surveys shared common attributes, there were certain differences among them, as outlined in Table 1.

**Table 1:** Comparison of studies conducted in the central Mamore River basin complex over a 20-year period. All surveys collect simple-platform data.

River	Date	# observers	Effort (Km surveyed)	Observer´s height above water level
**Ibare**	Jul/2014	4	75.10	4 m
	Aug/2019	4	71.53	
	Aug/2021	5	71.53	
	Jul/2022	3	71.53	
**Mamore**	Aug/1998	2	128.36	7 m
	Jul/2014	4	127.86	4 m
	Aug/2019	4	128.36	
	Jul/2022	3	128.36	
**Tijamuchi**	Aug/1998	2	185	3 m
	May/1999	2	185	3 m
	Jul/2014	3	169. 49	4 m
	Aug/2019	4	169. 49	

For each transect, a 12 m boat was used with a sighting platform approximately 4 m high located at the bow used by observers to count dolphins. A team of at least four observers/recorders (data recorder, sightings data recorder, and two observers) participated in the visual search for BRD using the naked eye. The observers were positioned at the front of the boat, providing a 180° field of observation ahead. Each sighting was recorded GPS position, group size, and landscape characteristics (type of both shores, habitat type, river width). Each team member rotated their roles, serving as an observer, a data recorder, or taking a resting position. These rotations occurred every two hours to alleviate observer fatigue and minimize perception bias. Observations were conducted within a consistent time frame, from 0700 to 1700 hours, with a one-hour break at midday. Furthermore, observations were made under favorable visibility conditions, characterized by low glare and the absence of rain or strong winds. The same team of trained observers, experienced in distance estimation and BRD sighting, participated in all the surveys.

The research permits allowing navigation and BRD surveys were granted by the Directorate of Biodiversity And Protected Areas of the Ministry of Environment and Water of the Plurinational State of Bolivia with the number: MMAYA/VMABCCGDF/DGABP/MEG N°0218/2022.

### Modeling approach

Trends in abundance of BRD were estimated within a generalized linear modelling (GLM) framework. Models with two different error structures: (a) Poisson and (b) negative binomial error distributions, both employing a logarithmic link function, were considered. The number of BRD counts was used as the response variable, while sampling year and river (Ibare, Mamore and Tijamuchi) were considered predictor variables. Additionally, the effort (kilometers traveled along the transects) was incorporated as a compensating factor in all models (an offset).

To explore potential non-linear relationships between sightings and temporal variables, the possibility of a quadratic dependence of year was also investigated. The best-fitting model was selected based on the lowest Akaike Information Criterion (AIC) value, with a difference in delta greater than 2 units. The DHARMA Package was used to evaluate model assumptions and to perform model selection [25]. The difference between factor levels was expressed as a percentage by calculating the exponential of each estimator from the best-fitting model. All models and statistical tests were performed using R Statistical Software [26].

## Results

Bolivian River Dolphin (BRD) direct counting from the 12 independent surveys (1998–2022) ranged from 11 to 362 individuals (Table 2, details within S1 Table).

**Table 2:** Number of Bolivian River Dolphin BRD (*Inia geoffrensis boliviensis*) observed in each survey.

River	Survey 1998	Survey 1999	Survey 2014	Survey 2019	Survey 2021	Survey 2022
**Ibare**	‐‐	‐‐	23	30	11	17
**Mamore**	126	‐‐	31	104	‐‐	42
**Tijamuchi**	289	231	157	362	‐‐	‐‐

A set of 12 GLM models were evaluated (each structure model attach in the S2 Table). The inference in trend was made using the 2nd best model because it contained year as one covariate (we are interested in describing trends in abundance) and, their delta AIC value (less the 2) indicated that was a well-supported model too [27]. This model assumed a negative binomial error distribution.

**Fig 2:**
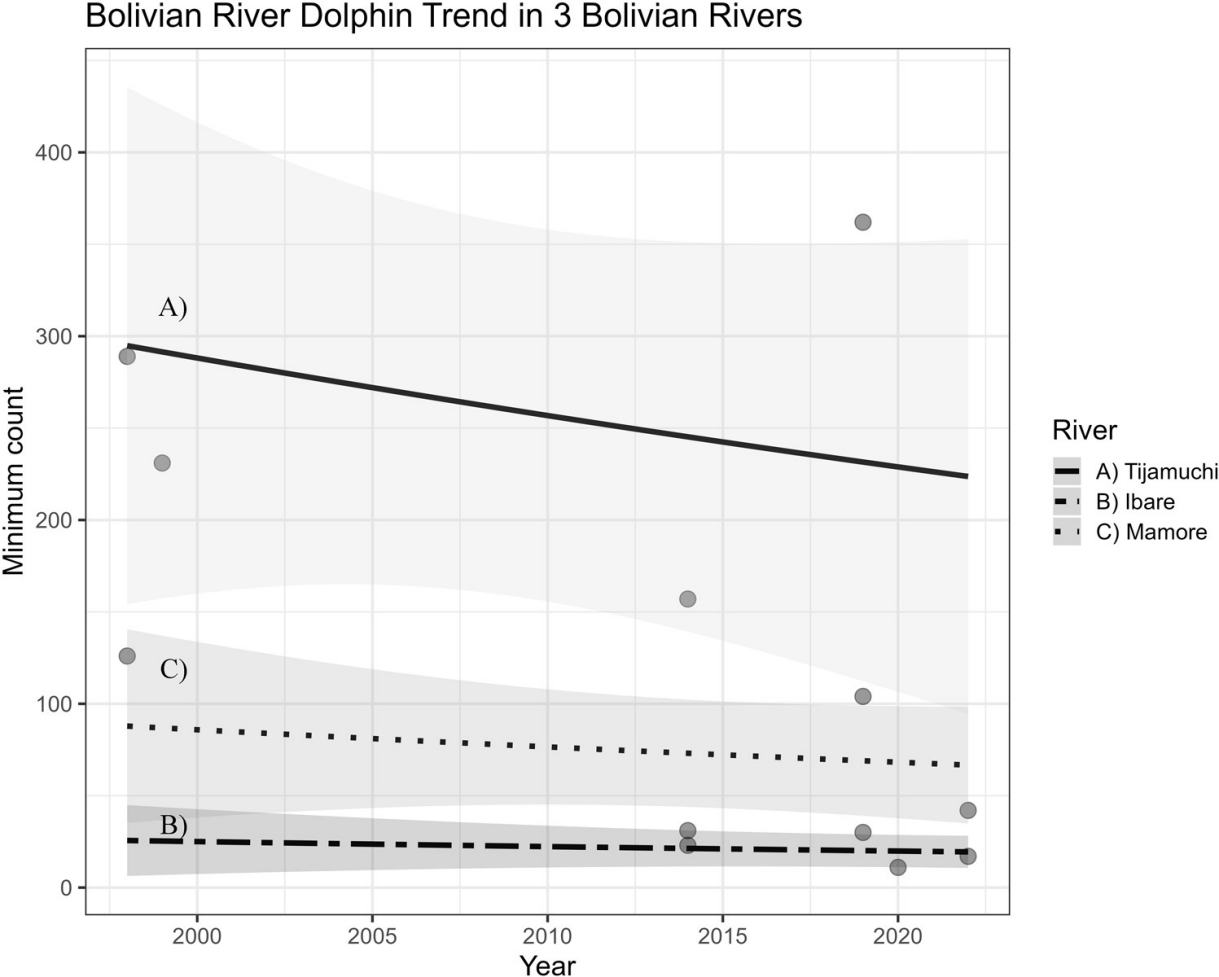
GLM best fitted model. Modeled trend for Bolivian River Dolphin populations in a complex Ibare-Mamore-Tijamuchi River System.

The best model fitted considered river and year as linear predictors without detecting any quadratic dependence. The mean estimate for the annual rate of population change was −0.0115 per year with a 95% range of -0.0396 to 0.0158 (p = 0.44, Table 3). While the model suggests a decline in the abundance of BRDs, the estimated trend is not statistically significant over the 24-year monitoring period. This apparent decreasing tendency is different among the evaluated rivers. The mean BRD count in the Ibare River is approximately 21.2% of the mean observed in the Tijamuchi River, while the mean BRD count in the Mamore River is 41.1% of the mean observed in the reference river (Table 3 and Fig 2).

**Table 3:** Model results for Bolivian River Dolphin (BRD) counts.

Parameter	Estimate	p-value
Year	-0.011511	0.44390
River-Ibare	-1.54819	6.49e-06***
River-Mamore	-0.88696	0.00273**
Explained deviance (R^2^)	86.65%	
Null deviance (d.f.^a^)	44.406 (11)	
Residual deviance (d.f.)	12.041 (8)	
Theta	6.59	

^a^ Degree Freedom

Significance: 0 ***;

0.001 **

## Discussion

According to a negative binomial distribution model, a significant decline in abundance of the Bolivian River Dolphin(BRD) in the central Mamore River basin during the 24-year study period is not yet evident. Although this area has been studied with a consistent monitoring approach by experienced observers, maintaining sampling consistency during the wet-dry transition season and using similar vessels, the trend remains unclear. However, the data in Fig 2 hints at the potential for a negative trend. This may be attributed to the sample size (n = 12) and/or high variability in the species distribution among transects due to environmental factors (such as zone productivity, river sinuosity) and intrinsic factors to the species like seasonal and ecological movements [28, 29]. Ongoing surveys in the region would prove immensely valuable in identifying any potential shifts in the likely small and geographically isolated BRD population.

Trends in abundance have been evaluated for the Amazonian River dolphin (*Inia geoffrensis*) in only two small geographical areas within its distribution: In the Amazon River in Colombia, Williams, Moore [30] but only comprised three different survey expeditions, although with sampling limitations they used a bootstrap regression and Bayesian model to estimate the probability of a conditional decline, concluding that there is insufficient evidence of population growth or decline for this species, but still suggested a declining trend. The second study in, the Brazilian Amazon, da Silva et al. [31] with 20-year data collection, concluded that the population in Central Amazon at Mamiraua Reserve surrounding has alarmingly decreased by half each decade.

Population trends are more readily observed in smaller areas, such as the 45 km of the Mamiraua lake system studied by da Silva et al. [31], considering that this species tend to have long-time site fidelity in other areas [32–34], as threats may have a more immediate impact in these localized regions. Furthermore, the time span covered by studies is often limited relative to the lifespan of long-lived species like cetaceans. Conducting large-scale surveys is costly and infrequent, resulting in coverage of only a portion of a population’s range. Consequently, changes in habitat distribution can lead to shifts in the proportion of the population available for sampling in a specific region [35]. Conventional modeling approaches applied to small cetacean studies struggle to differentiate whether apparent changes in abundance reflect shifts in population size or variations in distribution [36].

However, notable observations indicate a decrease in BRD populations in the Ibare and Mamore rivers by 21.2% and 41.1%, respectively, when compared to the Tijamuchi river (p<0.05). Previous reports by Aliaga-Rossel and Quevedo [37] and Aliaga-Rossel, Guizada-Duran [38] have also highlighted a noticeable decline in the minimum count of BRDs in both the Ibare and Tijamuchi rivers. The increase of boats, nets, and illegal overfishing activities, coupled with the decline in fish populations, are recurring trends consistently identified by local communities during the diagnostic assessments conducted in the area [22, 39, 40], potentially contributing to this decline.

The processes of extinction involve progressive decreases in abundance and geographic range, caused by both natural and human factors [41, 42]. While knowledge of population size and trend is fundamental for determining the risk of species extinction, it is not the only factor to consider. It is necessary to also contemplate factors such as geographic distribution, habitat fragmentation, pressure exerted by human activity, availability of food resources, and adaptive capacity to environmental changes. All these aspects must be carefully analyzed to gain a comprehensive understanding of the situation and to take appropriate measures for the conservation of the BRD and its habitat in Bolivia.

Furthermore, interviews conducted with local communities in the study area have revealed their perception of a consistent depletion of fishery resources over the years [40, 43]. This includes a reduction in the sizes of larger fish and increased difficulty in catching certain (commercial) species. Consequently, many fishermen perceive BRDs as their main competitors for resources and intentionally harm, kill them or use them as bait. Similar situations have been reported in Peru, Colombia, and Brazil [29, 31, 44].

Throughout the assessed period, extreme weather events, such as the 2008 drought and severe flooding in 2010 and 2014, may have significantly influenced population dynamics by necessitating the migration of numerous groups of river dolphins [22]. Although BRD populations exhibit a significant level of residency and limited large-scale movements [33], efforts to investigate migratory movements began in 2017 by Mosquera-Guerra et al. [45] but also in a limited area of Itenez River.

During the study period, the COVID-19 pandemic (2020–2021) had significant implications that could have influenced the presence and abundance of BRD due to the substantial reduction in human river usage. However, the strict lockdown in Bolivia lasted less than four months, and local activities by indigenous and riverside communities, such as fishing, did not cease. The survey conducted in 2020 in one of the study rivers showed no noticeable change in habitat use and population trends (Fig 2). Mosquera et al. [46] noted that home range and occupancy are influenced by factors such as sexual condition, ecological dynamics of prey (like abundance and movements), and the flood pulse’s impact on habitat use intensity. Therefore, during the pandemic, these predictors might not have been affected, unlike the Ganges River dolphin in India, where some changes in habitat use were observed, with dolphins approaching the riverbanks [11, 47]. Although the precise impact of the increasing threats on the population of BRDs remains uncertain, and the total trend is not statistically evident, we strongly believe that it is still necessary to continue with the monitoring the BRD population to account for populations and correlate this information with anthropogenic pressures, such as incidental and directed fishing-related catches. Similar patterns have been documented in neighboring countries [6, 18, 29].

The drastic declines in freshwater cetacean populations are not a new phenomenon. In Asia, the trend became critical starting in 1996 and resulted in the extinction of the Baiji or Chinese River dolphin (*Lipotes vexillifer*) only twelve years later by 2008 [9, 48]. Another critical case was the Ganges River dolphin, *Platanista gangetica*, whose populations began to decline since the mid-20th century due to the loss of habitat quality, human expansion, and finally by construction of barriers and dams, leading to its urgent classification as an "Endangered" species on the IUCN Red List since 1994 [8, 11]. Despite the historically perceived relative abundance of freshwater dolphins in South America, there is a growing concern regarding the reported impact of fishing-related mortality and the uncontrolled increase of human activities, as mentioned before. This, coupled with the limited distribution of the BRD, poses a significant risk to the overall well-being of the populations of this species in the not-so-distant future. Therefore, we strongly recommend large-scale and long-term conservation measures that generate essential data, which can drive the development of well-structured management programs and effective policy initiatives to prevent the subspecies from facing the same fate as Asian freshwater dolphins.

## Supporting information

S1 Table Minimal data set of BRD count in each survey. Minimum BRD counts in each survey used for modeling.
(DOCX)

S2 Table All alternative full GLMs. GLMs with number of BRD as response variable, and effort like offset, using two structure error (poisson and negative binomial).
(DOCX)
