# Supplementary material for: Bolivian River Dolphin trends: A long-term analysis in the Mamore basin
Source: PLoS One. 2024 Oct 4;19(10):e0308806. doi: 10.1371/journal.pone.0308806 (PMC11452032; doi:10.1371/journal.pone.0308806)
Supplement: S1 Table — Minimum BRD counts in each survey used for modeling. (DOCX) [file pone.0308806.s001.docx]

**SP 1 Table. Minimal data set of BRD count in each survey.** Minimum BRD counts in each survey used for modeling.

| River | Month | Year | Season | Effort-Km | BRD Count |
| --- | --- | --- | --- | --- | --- |
| Ibare | July | 2014 | Down | 75.10 | 23 |
| Ibare | August | 2019 | Down | 71.53 | 30 |
| Ibare | August | 2021 | Down | 71.53 | 11 |
| Ibare | July | 2022 | Down | 71.53 | 17 |
| Mamore | August | 1998 | Down | 128.36 | 126 |
| Mamore | July | 2014 | Down | 127.86 | 31 |
| Mamore | August | 2019 | Down | 128.36 | 104 |
| Mamore | Jul | 2022 | Down | 128.36 | 42 |
| Tijamuchi | August | 1998 | Down | 185 | 289 |
| Tijamuchi | May | 1999 | Down | 185 | 231 |
| Tijamuchi | July | 2014 | Down | 169.49 | 157 |
| Tijamuchi | August | 2019 | Down | 169.49 | 362 |
