# Supplementary material for: Bolivian River Dolphin trends: A long-term analysis in the Mamore basin
Source: PLoS One. 2024 Oct 4;19(10):e0308806. doi: 10.1371/journal.pone.0308806 (PMC11452032; doi:10.1371/journal.pone.0308806)
Supplement: S2 Table — GLMs with number of BRD as response variable, and effort like offset, using two structure error (poisson and negative binomial). (DOCX) [file pone.0308806.s002.docx]

**SP 2.** **Table. All alternative full GLMs.** GLMs with number of BRD as response variable, and effort like offset, using two structure error (poisson and negative binomial).

| Model | ΔAIC |
| --- | --- |
| River, Negative binomial error distribution | 0 |
| Year or River, Negative binomial error distribution | 1.34 |
| River or quadratic River, Negative binomial error distribution | 2.93 |
| Year and River, Negative binomial error distribution | 3.12 |
| River and quadratic River, Negative binomial error distribution | 4.39 |
| Year, Negative binomial error distribution | 11.17 |
| Year and quadratic River, Poisson error distribution | 25.75 |
| Year and River, Poisson error distribution | 110.61 |
| River or quadratic River, Poisson error distribution | 114.56 |
| River, Poisson error distribution | 145.69 |
| Year or River, Poisson error distribution | 147.63 |
| Year, Poisson error distribution | 496.99 |
